# Indonesian dengue burden estimates: review of evidence by an expert panel

**DOI:** 10.1017/S0950268817001030

**Published:** 2017-05-26

**Authors:** T. Y. M. WAHYONO, J. NEALON, S. BEUCHER, A. PRAYITNO, A. MOUREAU, S. NAWAWI, H. THABRANY, M. NADJIB

**Affiliations:** 1 Center for Health Economics and Policy Studies, Universitas Indonesia, Depok, Indonesia; 2 Sanofi Pasteur, Asia & JPAC Region, Singapore; 3 Department of Child Health, Faculty of Medicine, University of Indonesia, Cipto Mangunkusumo Hospital, Jakarta, Indonesia; 4 Sanofi Pasteur, Marcy L'Etoile, France; 5 Sanofi Pasteur, Jakarta, Indonesia

**Keywords:** Epidemiology, Delphi, dengue, Indonesia, under-reporting

## Abstract

Routine, passive surveillance systems tend to underestimate the burden of communicable diseases such as dengue. When empirical methods are unavailable, complimentary opinion-based or extrapolative methods have been employed. Here, an expert Delphi panel estimated the proportion of dengue captured by the Indonesian surveillance system, and associated health system parameters. Following presentation of medical and epidemiological data and subsequent discussions, the panel made iterative estimates from which expansion factors (EF), the ratio of total:reported cases, were calculated. Panelists estimated that of all symptomatic Indonesian dengue episodes, 57·8% (95% confidence interval (CI) 46·6–59·8) enter healthcare facilities to seek treatment; 39·3% (95% CI 32·8–42·0) are diagnosed as dengue; and 20·3% (95% CI 16·1–24·3) are subsequently reported in the surveillance system. They estimated most hospitalizations occur in the public sector, while ~55% of ambulatory episodes are seen privately. These estimates gave an overall EF of 5·00; hospitalized EF of 1·66; and ambulatory EF of 34·01 which, when combined with passive surveillance data, equates to an annual average (2006–2015) of 612 005 dengue cases, and 183 297 hospitalizations. These estimates are lower than those published elsewhere, perhaps due to case definitions, local clinical perceptions and treatment-seeking behavior. These findings complement global burden estimates, support health economic analyses, and can be used to inform decision-making.

Dengue is a systemic viral disease, transmitted to humans by the bite of infected *Aedes* spp. mosquitoes throughout the tropical and subtropical world. It results in substantial disease burden, health service disruption and costs [1]. Historically, the World Health Organization (WHO) estimated 50–100 million global infections per year including 500 000 dengue hemorrhagic fever (DHF) cases and 20 000 deaths but more recent modeling studies have found approximately four billion people in over 120 countries at risk, with 50–100 million annual symptomatic cases, mostly occurring in the Asia–Pacific region [2,3].

Indonesia has over 900 permanently inhabited islands extending over 5000 km from east to west. Since the first dengue reports in Jakarta and Surabaya in 1968, the disease has been expanding in incidence and geography, and is likely hyperendemic (i.e. multiple co-circulating serotypes) nationwide [4,5]. Notification of DHF is mandatory and Indonesia typically reports the highest number of cases in the WHO Southeast Asia Region [1]. Between 2001 and 2011, there was a reported average of 94 564 cases and between 472 and 1446 deaths per year [6]. The surveillance system uses WHO 1997 case definitions, whether clinically or laboratory diagnosed, but likely captures only a proportion of symptomatic disease due to inconsistent health-seeking behavior, non-specific symptoms, limited use of imperfect diagnostics and health systems issues. Between provinces, significant variation exists in reported incidence rates which may be a function of disease dynamics, surveillance and reporting practices, or both. Under-reporting is thus a complex product of geographical, clinical, epidemiological, laboratory, and health system factors. It may be that the introduction of point-of-care dengue rapid diagnostic tests has increased the reporting rate but data documenting this effect are currently lacking, and the full disease burden is unknown.

Estimating the public health and economic burdens of dengue are elements of the WHO *Global Strategy for Dengue Prevention and Control, 2012–2020*, and are priorities of many ministries of health to support disease control planning, allocation of resources and assessment of the value of novel prevention measures, including vaccination. Accordingly, a range of empirical or extrapolative methods have been employed to make more complete disease burden estimates in Indonesia and other countries [7]. However reliable data to make empirical assessments, particularly robust epidemiological data from active surveillance projects, are often lacking. A Delphi panel is a structured communication process which aims to achieve a convergence of opinion on a specific real-world issue. Experts make iterative estimates to answer a predefined research question, under the assumption that the range of answers will narrow as the process progresses. A group discussion makes each participant aware of the range of opinions and their rationale, information which is used to refine subsequent estimates, typically leading to confluence of opinion based on the expertise of the panel. The process is stopped upon reaching predefined criteria [8]. In combination with statistical methods, this approach has been used to calculate an overall adjustment factor for dengue under-reporting in the Philippines, Malaysia, and India, and it can be used to derive health system parameters which are otherwise unavailable [9,10].

A Delphi panel meeting was convened in Jakarta on 8 December 2015 comprising 14 experts, including infectious disease physicians and pediatricians, national specialists in dengue treatment guidelines and epidemiology, healthcare system managers, surveillance officers, academics, and laboratory workers, from different geographical areas across Indonesia, invited based on the advice of national-level dengue experts (full list of panelists provided in acknowledgments). The panel was expected to estimate the proportion of symptomatic dengue cases captured in the surveillance system and thus enable calculation of national-level dengue burden estimates. The panel also estimated the percentage of hospitalized and ambulatory dengue cases treated in private and public institutions.

A range of epidemiological and clinical data documenting current knowledge and gaps related to dengue in Indonesia was first presented to the panel, to align on recent study results and their methods. With the explanations that: (a) ‘dengue case’ refers to any patient whose symptoms are the result of infection with a dengue virus, including mild cases (e.g. fever >38°C for ⩾1 day) and those which present atypically; (b) ‘dengue diagnosis’ refers to a dengue case with a dengue diagnosis from a physician according to local practices (clinical and/or laboratory confirmation); (c) ‘healthcare facility’ refers to a licensed clinic, hospital, or other health provider (e.g. subdistrict-level primary healthcare center); and (d) ‘hospitalized dengue case’ is any dengue case spending at least one night in a healthcare facility; panelists were asked five questions, each of which was to be considered from the national perspective:

**Q1:** What percentage of dengue cases enters a healthcare facility to seek treatment?
**Q2:** Of all dengue cases entering a healthcare facility, what proportion is diagnosed as dengue?
**Q3:** Of dengue cases diagnosed in a healthcare facility what proportion is then reported in the routine Indonesian dengue surveillance system statistics?
**Q4:** Of dengue cases entering an Indonesian healthcare facility, what proportion is hospitalized for any duration?
**Q5:** Among all dengue cases entering healthcare facilities, what proportion is seen in the public sector if: (a) hospitalized; (b) outpatient (i.e. ambulatory).

Anonymous responses were collected by a moderator who aggregated the data, presented them to the group and facilitated a discussion. Participants were then invited to re-cast their votes in light of the previous results and discussions. The process was terminated after three rounds of voting (two rounds of discussion). Medians of final round votes were used for analysis, and a bootstrapping resampling method (200 samples; SAS software) employed to provide variability based on the theoretical non-parametric distribution of observed values, enabling estimation of medians and their 95% confidence intervals (CIs) [11]. These medians were used to calculate the total number of symptomatic dengue cases occurring in Indonesia via generation of an overall expansion factor (EF_O_):

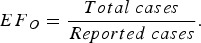


By proportionally adjusting a theoretical 100% of symptomatic cases according to the responses to the questions above, this can be logically calculated according to the formula:

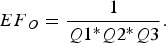


The total number of cases can be estimated as:

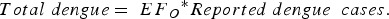


Cases could be further stratified into hospitalized and ambulatory dengue, which are likely under-reported in different magnitudes and incur different public health consequences and costs:

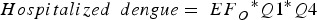

and

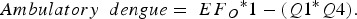


Specific EFs for hospitalized/ambulatory dengue EF_H_ and EF_A_ are often reported [7,9]. While they do not affect final burden estimates here, they may have value for policy-makers and can be calculated assuming the proportion of hospitalized and ambulatory cases within dengue-reporting systems is known by:

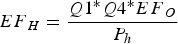
and

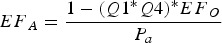

where *P*_h_ and *P*_a_ are the proportion of reported cases which is hospitalized/ambulatory (*P*_h_ = *100* *−* *P*_a_). Based on local experience that most reported cases are hospitalized, we made a base-case assumption of *P*_h_ = 90%, with uncertainty assessed by applying rates from 80% to 99% in sensitivity analysis.

Proportions of cases seen in public/private facilities were similarly adjusted using the responses to question 5. National-level estimates were calculated by multiplying these EFs by the number of reported dengue cases in Indonesia, from 2006 to 2015 [12].

One participant departed after the first voting round leaving 13 voting participants at the meeting. At the third vote, four questions were unanswered leaving a total of 74 responses in the analysis. There was significant confluence of opinion by the third round with more than half (45/74) of votes agreeing on the response to each question. Voting summaries from the final round, and median estimates from bootstrapping resampling and their 95% CIs are provided in Table 1. Panelists estimated that, of all symptomatic dengue episodes, 57·8% (95% CI 46·6–59·8) enter healthcare facilities to seek treatment; 39·3% (95% CI 32·8–42·0) is diagnosed as dengue; and 20·3% (95% CI 16·1–24·3) is subsequently reported. In all, 31·5% (95% CI 24·4–35·5) of cases are hospitalized. Of all cases entering the healthcare system, 20·0% (95% CI 14·5–24·2) are hospitalized in the public sector (with a public/private split in hospitalized cases of 64%/36%) and 12·0% (95% CI 9·8–14·1) are outpatients in the public sector (public/private split in ambulatory cases: 45%/55%).

**Table 1.** Summary final results of the Delphi panel and derived medians and their 95% confidence intervals following boot-strapping resampling

Question	Number of votes	Mean (%)	Mode (%)	Median response (%)	Bootstrap-derived median (95% CI)
1. What percentage of dengue cases enter a healthcare facility to seek treatment?	13	57	60	60	57·8 (46·6–59·8)
2. Of all dengue cases who enter a healthcare facility, what proportion is diagnosed as dengue?	13	70	70	70	70·0 (69·6–70·5)
3. Of dengue cases diagnosed in a healthcare facility what proportion is then reported in the routine Indonesian dengue surveillance system statistics?	12	54	60	60	56·7 (50·7–59·9)
4. Of dengue cases who enter an Indonesian healthcare facility, what proportion is hospitalized for any duration?	12	56	60	60	59·0 (55·0–60·1)
5 (a). Among all dengue cases who enter healthcare facilities, what proportion is seen in the public sector if hospitalized?	12	68	70	70	68·6 (65·6–70·0)
5 (b). Among all dengue cases who enter healthcare facilities, what proportion is seen in the public sector if outpatient?	12	53	60	52·5	50·9 (50·8–51·0)

These estimates gave rise to an EF_O_ of 5·00 (95% CI 4·11–6·21); EF_H_ of 1·66 (95% CI 1·51–1·86), and EF_A_ of 34·01 (95% CI 27·85–44·72) and, when combined with passive surveillance data, a 2006–2015 annual average of 612 005 symptomatic cases (Fig. 1). This varied from a low of 328 704 in 2011, to a high of 790 770 in 2007. This equates to a total from 2006 to 2015 of 3 537 238 (95% CI 2 854 797–3 657 332) cases entering health facilities; 2 476 067 (95% CI 1 986 082–2 577 322) dengue diagnoses and 1 832 969 (95% CI 1 665 785–2 052 687) hospitalizations, 1 164 543 of which are seen in the public sector. Varying the hospitalization rate from 80% to 99% led to EF_H_ ranges of 1·51–1·87 and EF_A_ from 17·01 to 340·13. As these rates are components of burden calculations they make no difference to final estimates here, but this variability emphasizes the importance of consistent assumptions and accurate methodological reporting.

**Fig. 1.**
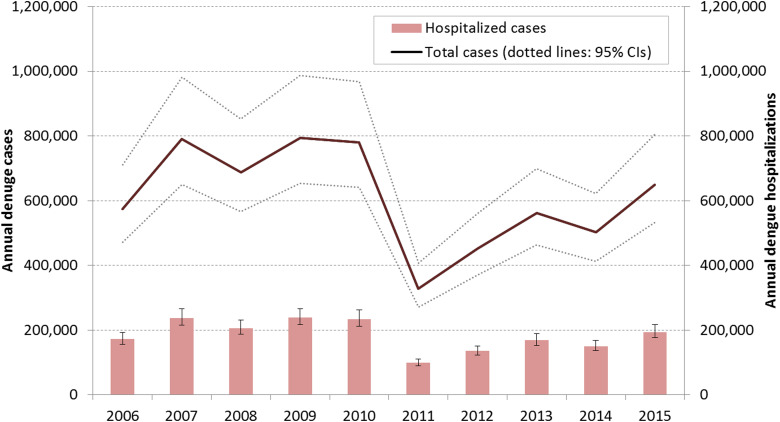
Estimated annual number of dengue cases and hospitalizations in Indonesia following adjustment of surveillance reports with EFs, and their 95% confidence intervals (CIs), 2006–2015.

These findings support previous reports that dengue is significantly under-reported in Indonesia, and provide granularity which was previously lacking, for example the finding that approximately 1/3 of all symptomatic cases is hospitalized for some duration. However, the magnitude of under-reporting is relatively modest in comparison with other studies: a regional analysis extrapolating from neighboring countries found an overall EF of 7·6 [7] and an analysis of published data found national under-reporting in Indonesia from 36- to 126-fold [13]. More recently, two prospective comparisons between active and passive surveillance systems have been published: a factory-based dengue cohort in West Java identified a dengue incidence rate of 17·3 cases/1000 person-years, 43-fold higher than rates recorded in the passive surveillance system; [14] and a comparative reanalysis of placebo arm data from a dengue vaccine clinical trial in Jakarta, Bandung, and Bali identified an overall EF of 11·5 [15]. Finally, two influential global dengue burden studies using complementary approaches based on dengue occurrence data, incidence rates from published cohorts, or vital registration and verbal autopsy estimated national burdens from which Indonesian EFs of 57 and 106, respectively, can be derived [2,3]. These recent studies are consistent in finding that dengue is significantly under-reported in Indonesia at magnitudes in significant excess of these Delphi panel estimates.

Dengue causes a spectrum of clinical disease and incidence rates are determined by the surveillance system and case definitions applied to describe symptomatic episodes. The experts participating in this Delphi panel, who are mostly familiar with dengue episodes requiring medical intervention, may be familiar with more severe and less frequent presentations of dengue than considered in other analyses, a possible explanation for these conservative projections [2]. Supporting this hypothesis, our estimates are similar to those from a 2013 paper (which found 792 829 annual cases), conducted before contemporary estimates were available [7]. Additionally, only dengue cases meeting a DHF case definition are notifiable in Indonesia, a probable reason why the nationally reported incidence rates are lower than those from neighboring countries [data not shown]. A recent analysis clearly described a relationship between clinical severity and under-reporting, it therefore remains important for policy-makers to understand methodological study aspects, case definitions, and their implications [15]. Simple comparisons between countries are rarely justified. Some of these observations are limitations of an expert-based approach and are reflective of local expert opinion. However, such a method enables exploration of experimentally challenging research topics in complex countries, understanding of expert views and their rationale, and projection of local experience and data to inform decision-making at the national level.
